# Dual dual-use research of concern: Publish *and* perish?*

**Published:** 2011-01

**Authors:** K. Satyanarayana

**Affiliations:** Editor, IJMR kanikaram_s@yahoo.com

New life sciences discoveries reported in the public domain have widely considered to have helped us in myriad ways: protecting public health, multiplying agricultural yields, fostering technological development and economic growth, and enhancing global stability and security. Scientists typically report their research in learned journals and strive hard for widest dissemination to potential users. As the renowned nuclear physicist Oppenhiemer said.“It is not possible to be a scientist unless you think that it is of the highest value to share your knowledge, to share it with everyone that is interested” and that …“Secrecy,”… “strikes at the very root of what science is, and what science is for”^1^. He was referring to the publication and dissemination of research and development of nuclear fission and the chain reaction that could (and did) led towards the creation of weapons of mass destruction (WMD). Nuclear science and technology continues to be the most closely guarded all over the world.

Life science researchers, especially in the area of biotechnology, have been somewhat in a similar situation: their discoveries have tremendous beneficial impact on health and agriculture but some have equally potential application for harmful use^2^. While such dual use of research has been known for long with even bio-weapon programs in some countries^3^, it is the anthrax attacks in the USA and elsewhere in 2001 that brought the threat of bioterrorism to the centre-stage. This menace is considered more serious and real than nuclear weapons research as the production of biological weapons is relatively easy and inexpensive. Compounding the problem, most information, data and tools to produce biological WMD are readily available in public domain.

Understandably, the post-2001 years spurred a series of events, actions and debates on the dual use research of concern (DURC). Dual-use biological research is defined as “biological research with legitimate scientific purpose that may be misused to pose a biologic threat to public health and/or national security”^4^. *i.e*., where products, equipments or ideas may also cause illness, death, panic or disruption in social life. In other words, any research that will enable and/or result in (*i*) eventual weaponization; (*ii*) severity of disease/symptoms; and (*iii*) mass casualty^5^.

Post 9/11, the first substantive debate on this issue was triggered off by the publication of a series of papers that appeared in 2001-02 with serious DURC. In the first study, researchers from Australia inserted the mouse IL-4 gene into the mousepox virus with the aim of sterilizing the mice as a means of pest control. But the result was a mousepox that became so virulent that it just killed the mice that were naturally resistant to and vaccinated against, ordinary mousepox^6^. This ‘super strain’ of mousepox is typical DURC: can be used to produce vaccine for vaccine-resistant smallpox but also as a bioterror agent. With no known treatment for smallpox and vaccination, our only defense long since stopped, such a strain of virus could be a very serious threat.

In the second study, ‘live’ polio virus was artificially synthesized from scratch - pieced together using a map of the polio virus RNA genome available over the Internet with strands of DNA obtained via mail-order^7^. Addition of a protein activated the synthetic virus killing infected mice^7^. This is both an exciting, innovative methodological research but a similar virus could be used as a potential WMD^7^.

In yet another study, researchers used DNA sequences available in public domain to engineer a protein, smallpox inhibitor of complement enzymes (SPICE) produced by the smallpox virus^8^. The research could lead to new treatments or vaccines both to immunize against the naturally occurring smallpox virus and to counteract the genetically modified strain but could help create a highly virulent vaccinia virus as well.

Given the implications of these papers for potential malevolent use with such ease, there were calls for a complete ban publication of such research or at least enforce partial ‘censorship’ of manuscripts like pruning the Methods section^9^. The researchers and the editors were clearly unwilling to modify the manuscripts. What is more, they strongly and expectedly defended their actions claiming that the benefits of publication of such research outweigh the risks, and that suppression of information in the Methods section is contrary to the tenets of scholarly communication, a view unsurprisingly shared by most scientists^10^.

At about the same time, the American Society for Microbiology (ASM), urged the US National Academy of Sciences (NAS) to take a lead in addressing the issue of DURC. The NAS held a two day meeting with scientists, security experts, journal editors and publishers, and government officials. The meeting resulted, among others, the first “Statement on Scientific Publication and Security” simultaneously published by *Science, Nature*, the *Proceedings of the National Academy of Sciences (PNAS)*, and the American Society for Microbiology journals^2^. There was consensus that DURC is indeed a serious and complex issue and the risk of open publication helping malevolent people real that needs to be addressed. The participants agreed on the following in respect of publication of DURC:^2^.

- Scientific information published in peerreviewed research journals must contain sufficient details to permit reproducibility as independent verification is essential for scientific progress including national security.
- The concern of potential abuse of published information is legitimate and therefore editors should deal responsibly and effectively in respect of safety and security issues in manuscripts submitted for publication.
- Scientists and editors should put in place appropriate levels of peer evaluation and design processes to accomplish effective review of systems of manuscripts with DURC and ensure timely implementation of such processes.
- Where the potential harm of publication outweighs the potential societal benefits, editors should consider modification of such a paper or even refuse publication. While all systems of widest dissemination of information such as seminars, workshops, web postings etc. that maximizes public benefits should be encouraged, where there is potential for misuse, scientists should exercise adequate caution in dissemination.

Meanwhile, the absence of national or international review or oversight bodies with self-governance responsibility much less legal authority to evaluate proposed research of DURC in terms of risks associated or the anticipated benefits was recognized. This led to setting up a Committee on Research Standards and Practices to Prevent the Destructive Application of Biotechnology under the chairmanship of Gerald Fink of Massachusetts Institute of Technology by the US Government. The Fink Report^11^called for, among others, increased education of the scientific community about the dual-use dilemma, expanded role of institutional biosafety committees to review research proposals for dual-use risks (as well as environmental dangers), self-governance of the scientific community (as opposed to governmental censorship) in matters related to publication of dual-use research findings and the establishment of a new advisory board to provide guidance to the government regarding the oversight of dual-use research.

Significantly, the Committee unambiguously favored self-governance by scientists and scientific journals to review publications with potential DURC and opposed any formal regulation by government. The Fink Committee also suggested that the National Security Decision Directive 189 (1985) which ensures unrestricted access to results of fundamental research to the maximum extent possible, should continue to be the basis for U.S. policy. As part of recommendations of the Committee^11^, the US Department of Health and Human Resources (DHHS) established the National Science Advisory Board for Biosecurity (NSABB)^4^ in 2004 with a 12 point charter that included, among others, the setting of working groups for developing criteria for many issues on DURC including dissemination of information.

Even before the first meeting of the NSABB, the system was put to test. The journal *Science* received a paper on ‘reconstructed’ Spanish Flu virus bearing all the identified gene sequences of the 1918 virus using the synthetic genomic approach^12^. The Spanish Flu virus killed about 100 million people in 1918-19, most serious pandemic recorded in human history. These researchers put together the virus based on the full sequence of the highly virulent strain of 1918 influenza published in *Nature*^13^ about the same time. As mandated by the law, the US HHS Secretary sought guidance of the NSABB^14^. The NSABB considered that the scientific benefit of the information being reported outweighed the potential risk of misuse and therefore unanimously recommended publication of the study. The paper duly appeared with an accompanying editorial discussing the potential biosecurity implications of the research^15^. Yet, *Science* Editor-in-Chief Donald Kennedy persuasively argued that even if the NSABB were to refuse permission, he would have gone ahead with its publication^16^. This is perhaps, the first formal referral to the NSABB by the HHS Secretary in respect of publication of DURC. Around the same time, a manuscript on the potential impact of contaminating the milk supply with botulinum toxin was submitted to the PNAS^17^. The DHHS, the nodal government agency charged with the responsibility of overseeing the DURC in the US, opposed publication of the PNAS paper, which the Journal eventually published^17^.

How serious is the problem of publication of DURC? Not of great concern, it appears, from available publication data. For example, of the 74000 biology papers received by *Nature* and the Nature group of journals during 2004-08, just 28 papers were considered of dual use concern^18^. It was about one paper per year at the *Science* and *PNAS* and 1-2 per year at the ASM journals^18^. Significantly, no paper has so far been rejected on grounds of biosecurity risk at any journal^18^. The manuscript on mathematical modeling of a terrorist attack on the food supply^17^was only delayed at the intervention by the US government^19^.

These events rekindled the devide in respect of disclosure of DURC between scientists and science editors on one side with regulators and some ethicists who seriously questioned the role and ability of those who generate and disseminate such information to address this issue seriously, on the other. The debate, however, sensitized scientists of the seriousness of the threat of DURC with several guidelines and codes of ethics brought out by professional bodies^20^. These guidelines clearly focused only on education and sensitization rather than legislation or other ‘top-down’ approaches^4^,^5^,^11^,^21^. Polices on publication were also clearly articulated in some reports^11^,^21^. The Royal Society Report^21^, for example, very strongly supported the view of scientists that preventing publication of basic research would not be really of much help as such scientific information can eventually get disseminated either through other journals, websites or conference proceedings *etc*. or someone else could easily repeat the research. Following the Statement^2^, journals like *Nature, Science*, PNAS and the ASM journals quickly established additional review systems for DURC by biosecurity experts^18^ that are also regularly being revised. However, journals from countries like Russia, China^22^ and India do not have such policies on publication of DURC. However, in India, DURC is receiving attention^23^; the Indian Society for Medical Microbiology devoted a full session to this issue in its 2010 Annual meeting. The *Indian Journal of Medical Research* will soon call for a meeting of Indian medical journal editors to formulate policy guidelines for publication of DURC.

There is still some apprehension that self-regulation will not work^9^,^24^ as the existing policies are primarily driven by and centred around scientists and science editors. Critics also questioned the efficacy of these policies as scientists do not understand security issues and have a conflict of interest in publishing^9^,^24^. Regulators and security experts, on the other hand, may not understand science adequately for an unbiased assessment. An inclusive oversight structure with security experts, legal experts, social scientists, ethicists, government, regulators and journal editors and civil society perhaps will be required. Areas as engineering, social sciences *etc*. should also be included^9^,^24^ in view of papers as mathematical modeling of potential impact of contaminating the milk supply with botulinum toxin^17^, essentially conducted by non-life scientists. The area of DURC it self, is still new and evolving and the experience gained is still inadequate, never seriously tested except perhaps once in the PNAS paper^17^. Also, if the experience on setting up of effective oversight systems in ethical conduct and reporting of biomedical research is any indication, scientists may not be able to put their house in order. It needed the intervention of a politician (the tenacious US Congressman John Dingle) to intervene and put in place appropriate legal and other systems that have significantly contributed to the sanitizing of science in the US, and elsewhere.

Clearly, in the current context the issue of DURC should be addressed in its entirety: (*i*) funding of research, (*ii*) access to research materials/tools; and (*iii*) access to means of making use through data/information in public domain. But there are some fundamental issues that need attention. Like there is still no clarity and consensus on what constitutes DURC itself^20^,^25^ underscoring the complexity of the issue, and associated problems. But the real challenges would continue to be governance and enforcement^9^,^19^,^20^,^25^ as scientists are clearly averse to any external intervention even while they agree for the need to have a formal structure to examine DURC. But continued resistance of the scientific community may not be prudent in the long run as a set of rules could well be enforced unilaterally^25^. The dilemma of DURC is best summed up by this Buddhist temple saying: “To every man is given the key to the gates of heaven. The same key opens the gate of hell”^9^. It is for scientists to take the call.
